# Indications and outcomes of rivaroxaban use in cats

**DOI:** 10.3389/fvets.2025.1561003

**Published:** 2025-06-20

**Authors:** Ella Yarsley, Claire R. Sharp, Corrin J. Boyd, Joonbum Seo, Erin Mooney

**Affiliations:** ^1^School of Veterinary Medicine, Murdoch University, Murdoch, WA, Australia; ^2^Centre for Terrestrial Ecosystem Science and Sustainability, Harry Butler Institute, Murdoch University, Murdoch, WA, Australia; ^3^Animal Referral Centre (ARC Central), Auckland, New Zealand; ^4^School of Veterinary Science, Massey University, Palmerston North, New Zealand; ^5^Small Animal Specialist Hospital, North Ryde, NSW, Australia

**Keywords:** thromboprophylaxis, aortic thromboembolism, anticoagulant, thrombus, immune-mediated haemolytic anaemia, clopidogrel, Consensus on the Rational Use of Antithrombotics in Veterinary Critical Care

## Abstract

**Introduction:**

The use of rivaroxaban, an oral direct factor Xa inhibitor, has only been described in a small number of publications in cats. The study objective was to describe the use of rivaroxaban in a large population of hospitalised cats.

**Methods:**

Cases were retrospectively identified from June 2017 to July 2024 at seven veterinary specialty hospitals. Any cat prescribed rivaroxaban was eligible for inclusion. Data extracted from the medical records included signalment (age, sex, breed), body weight, reason for commencing rivaroxaban, dose and duration of rivaroxaban, concurrent anticoagulant and antiplatelet therapies, potential rivaroxaban adverse effects, and outcome. Non-parametric descriptive statistics are reported.

**Results:**

In total, 66 cats were included. Median rivaroxaban dose was 2.5 mg (Min-Max 1.25–10, Q1-Q3 2.5–5.0), equal to 0.73 mg/kg/day (Min-Max 0.28–1.87, Q1-Q3 0.53–1.0). A total of 36 cats (54.5%) were within the suggested dose range of 0.5–1 mg/kg/day of the Consensus on the Rational Use of Antithrombotics in Veterinary Critical Care (CURATIVE) guidelines, 14 (21.2%) were below, while 16 (24.2%) were above. Median duration of rivaroxaban was 26.5 days (Min-Max 0–442, Q1-Q3 2–60), although followup was variable. The indication for rivaroxaban administration was confirmed thrombosis (48, 72.7%), strong clinical suspicion of thrombosis (6, 9.1%), and prophylaxis (12, 18.2%). Most thrombi were arterial, including aortic thromboembolism affecting both pelvic limbs (25/54 cats with thrombosis, 46.3%), arterial thrombosis affecting a single limb (16, 29.6%), and cardiac chamber thrombus (7, 13%). Cardiac disease was the most common thrombosis risk factor (53/66, 80.3%). Other CURATIVE defined risk factors included immune-mediated haemolytic anaemia in four cats (6.1%) and sepsis in one cat. Other thromboprophylaxis administered included clopidogrel in 58 cats (87.9%), dalteparin in 8 cats (12.1%), and aspirin in 4 cats (6.1%). Potential adverse effects prompting rivaroxaban discontinuation included one case each of vomiting, a cerebrovascular accident, gastrointestinal bleeding, and haemorrhagic pleural effusion. Forty-five cats (68.2%) survived to hospital discharge, 14 (21.2%) were euthanised, two (3%) died, and five (7.6%) were taken home against medical advice.

**Conclusion:**

Rivaroxaban was well tolerated in a large population of cats, predominantly prescribed for arterial thrombosis associated with cardiac disease.

## 1 Introduction

Thromboprophylaxis has become an important medical consideration in small animal practice given increasing understanding of the risk of thrombosis associated with certain disease states and advances in thromboprophylaxis medications (1). Awareness of the evidence to support thromboprophylaxis has been aided by the publication of the American College of Veterinary Emergency and Critical Care Consensus on the Rational Use of Antithrombotics in Veterinary Critical Care (CURATIVE) guidelines in 2019 (2). These evidence-based guidelines describe the risk of dogs and cats developing thrombosis and recommend appropriate medical management with anticoagulants and antiplatelet drugs (2–9). Cardiomyopathy is the primary disease conferring a high risk of developing thrombosis in cats, particularly aortic thromboembolism (ATE), and as such recommendations for thromboprophylaxis have also been included in the ACVIM consensus statement guidelines for the classification, diagnosis, and management of cardiomyopathies in cats (10). Specifically, the ACVIM guidelines recommend that, in addition to the antiplatelet agent clopidogrel, anticoagulant treatment using low-molecular weight heparin (LMWH), unfractionated heparin (UFH), or an oral FXa inhibitor, such as rivaroxaban, should be started as soon as possible in cats with cardiogenic ATE, albeit denoting a low level of evidence (10).

Factor Xa (FXa) is a desirable pharmacological target for thromboprophylaxis as it catalyzes the production of large amounts of thrombin (11). Approximately one molecule of FXa can result in the production of up to 1,000 molecules of thrombin (12). Rivaroxaban is a direct oral FXa inhibitor, which selectively and directly inhibits FXa, blocking this thrombin burst (12). Owing to insights gained from randomised controlled trials (RCTs) in human beings, the use of rivaroxaban for prevention and treatment of thrombosis is included in guidelines for subsets of human patients with acute coronary syndromes (alone or as part of dual therapy) (13), atrial fibrillation (14), peripheral arterial disease (15), cancer (16–18), and COVID-19 (19). Rivaroxaban is generally considered safe and efficacious in humans, with the convenience of daily or twice daily dosing, without routine requirement for therapeutic monitoring (11, 20, 21).

Only a single paper on the use of rivaroxaban in healthy cats had been published at the time that the CURATIVE guidelines were written (22), but nonetheless, reference to direct Xa inhibitors was included. Specifically, it was stated that no evidence-based recommendations could be made regarding the use of direct Xa inhibitors over UFH, LMWH, or warfarin, in cats. Nonetheless, it was suggested that the use of direct Xa inhibitors could be considered in cats based on reliable pharmacokinetics (PKs) and a favorable preliminary safety profile and that direct Xa inhibitors should be used in preference to warfarin (2). A dose of 0.5–1 mg/kg per day was suggested in cats based on the aforementioned single pharmacokinetic (PK)–pharmacodynamic (PD) study in healthy cats (22).

To date, only five *in vivo* studies have described the use of rivaroxaban in cats (22–26). Despite ongoing research in this field since development of the CURATIVE guidelines, including recent RCTs of rivaroxaban use in cats, current studies focus on outpatient cardiac indications (23–26), with little evidence of its general use in hospitalised cats with a variety of clinical diseases. In an attempt to begin to fill this knowledge gap, the objective of this study was to describe the clinical use of rivaroxaban in hospitalised cats. We hypothesised that rivaroxaban would be used most commonly for cats with thrombotic complications secondary to a variety of diseases, as opposed to prophylactically, and that few cats would have side effects attributable to rivaroxaban.

## 2 Materials and methods

### 2.1 Data collection

Cases for this historical case series were included from seven hospitals in Oceania; The Animal Hospital at Murdoch University (TAHMU) in Western Australia, two hospitals in the Animal Referral Centre (ARC) network in Auckland New Zealand, and four hospitals in the Small Animal Specialist Hospital (SASH) network (North Ryde, Western Sydney, Alexandria, and Central Coast, all in New South Wales). Cases at TAHMU were retrospectively identified with a fee code search of electronic medical records system (RxWorks), for cats billed for “Rivaroxaban 15 mg tablet” from June 2017 to August 2024. A single fee code for the 15 mg tablets was used because this was the only size rivaroxaban tablet stocked at TAHMU during the study period. Any cat prescribed rivaroxaban was eligible for inclusion. For patients of the ARC and SASH, cases were identified by searching the medication section of patient files within the electronic medical record system (Ezyvet) between June 2017 and August 2024. Cats for whom rivaroxaban was dispensed from the hospital pharmacy, or an external prescription was provided, were included. Cases were excluded if the medical records regarding the use of rivaroxaban were incomplete. Duplicates were removed if rivaroxaban was dispensed multiple times within the same course of treatment. If a patient received multiple treatment periods of rivaroxaban, interrupted by time not receiving rivaroxaban, each treatment period was considered separately.

The medical records for each cat were reviewed, and information was recorded into a standardised data collection tool using Research Electronic Data Capture (REDCap) (27, 28). Information recorded included date of birth (DOB), breed, sex, weight, start date of rivaroxaban, last known date of rivaroxaban use, dose administered, frequency of administration, and if there were any dose adjustments. Rivaroxaban dose was also classified based on whether it was below, within, or above, the dose range suggested by the CURATIVE guidelines for cats of 0.5–1 mg/kg/day (5). Age was calculated within REDCap as the difference between the start date of rivaroxaban therapy and the DOB. The last date of rivaroxaban use was based on communication with the client and/or the duration prescribed on the last prescription provided. Duration of rivaroxaban therapy was calculated within REDCap as the difference between the date of the first and last doses of rivaroxaban. As such a cat that received only a single dose of rivaroxaban had a duration of therapy listed as 0 days. Indication for rivaroxaban use, evidence of thrombosis and type of thrombosis, as well as any potential or confirmed side effects were documented. Information regarding other thromboprophylaxis was also recorded, including the specific drugs and whether it was started before, during, or after rivaroxaban therapy. Evidence of thrombosis was classified as confirmed, or a strong clinical suspicion of, thrombosis. Confirmation of thrombosis was achieved through direct visualisation either with ultrasonography (US), computed tomography (CT) with intravenous contrast, or magnetic resonance imaging (MRI). Aortic thromboembolism to the distal limbs could also be confirmed by identification of pathognomonic clinical signs, notably an acute onset of pain, paresis, pulselessness, pallor, and poikilothermy (29). Strong clinical suspicion of thrombosis was determined through physical examination, history, and clinicopathologic and diagnostic imaging results in the absence of thrombus visualisation and was based on expert review of medical records by the authors (CRS, CJB, JS, and EM). Indications for rivaroxaban use were primarily classified according to the CURATIVE guidelines (2, 3). Specifically, two conditions were considered major risk factors for thrombosis according to CURATIVE: (i) heart disease with left atrial enlargement, spontaneous echocardiographic contrast, and/or reduced left auricular appendage flow velocity and (ii) arrhythmias and structural cardiac disease (3, 8). Cats that presented with arterial thromboembolism that did not have a complete echocardiogram by a cardiology or radiology specialist were also considered to have a major CURATIVE risk factor if they had any evidence of cardiac disease, such as a heart murmur, gallop sound, cardiomegaly on thoracic radiographs, and/or concurrent clinical or radiographic signs of congestive heart failure. In addition, cats with a hypertrophic cardiomyopathy phenotype on an echocardiogram without the aforementioned conditions, but with left auricular enlargement and/or reduced left auricular flow velocity, were also considered to have cardiac disease as a major CURATIVE risk factor. Other risk factors for thrombosis in cats identified in the CURATIVE guidelines included immune-mediated haemolytic anaemia (IMHA), heartworm disease, sepsis, protein losing diseases (nephropathy and enteropathy), sepsis, and surgical correction of congenital portosystemic shunt (3, 8). In addition, indications not included in the CURATIVE guidelines that prompted clinicians to administer rivaroxaban for thromboprophylaxis were recorded. Cases with potential or confirmed rivaroxaban side effects were based on expert review by four of the authors (CRS, CJB, JS, and EM). Finally, concurrent diseases and case outcome data were also recorded. Outcome of the initial hospitalisation period was recorded as survived to discharge, taken home against medical advice, died naturally, or euthanised. When a patient was a non-survivor, it was recorded as died from the primary disease rivaroxaban was used for, a comorbidity/other, or thrombotic complication.

### 2.2 Statistical analysis

Data were assessed for normality by visual inspection. Descriptive statistics were generated by the REDCap reporting function. Non-normally distributed continuous data are reported as median (Min-Max, Q1-Q3). Categorical data are reported as number, proportions, and percentages.

## 3 Results

### 3.1 Population and rivaroxaban dosing

The search strategies identified 66 cats that had received rivaroxaban. Summary statistics of cat demographics, rivaroxaban dosing, and outcome are included in Table 1. All but two cats received once daily rivaroxaban dosing. With regard to dose relative to the CURATIVE recommendations, 36 cats (54.5%) were within the suggested dose range of 0.5–1 mg/kg/day, 14 (21.2%) were below, while 16 (24.2%) were above. The majority of cats stayed on the original dose of rivaroxaban for the duration of their therapy (59, 89.4%), with seven cats (10.6%) having their dose adjusted. Of these, four cats had their dose increased due to evidence of thrombosis: Three had been on 2.5 mg PO SID initially and were increased to 2.5 mg PO BID, while the other was on 5 mg PO SID pm initially, and an additional 2.5 mg PO was added as an am dose. Reasons for dose adjustment in the three other cats included dose reduction as part of a taper, to conform to CURATIVE guidelines where the previous dose was not within the recommendations (1.25 mg PO SID increased to 2.5 mg PO SID) and was not specified in the remaining cat. Reasons for rivaroxaban discontinuation include non-survival (31, 47%), lost to follow-up (20, 30.3%), adverse effects attributed to rivaroxaban (2, 3%), end of the scheduled course (2, 3%), being switched to an alternative thromboprophylaxis plan (2, 3%), and difficulty with administration of oral medication (2, 3%). Six cats (9.1%) were still receiving rivaroxaban at the time of study data collection, and in one case, the reason for rivaroxaban discontinuation was unclear from the medical record.

**Table 1:** Summary statistics of cat demographics, rivaroxaban dosing, and outcome, for 66 hospitalised cats prescribed rivaroxaban.

Parameter	Summary statistics
Treating hospital	
SASH North Ryde	25, 37.9%
The Animal Hospital at Murdoch University	22, 33.3%
Animal Referral Centre, Auckland	6, 9.1%
SASH Western Sydney	6, 9.1%
SASH Alexandria	5, 7.6%
SASH Central Coast	2, 3%
Age (years)	
Median	7
Q1-Q3	4.8–11.51
Min-Max	1.0–20.36
Sex	
Male neutered	41, 62.1%
Female spayed	24, 36.4%
Male intact	1, 1.5%
Breed	
Domestic shorthair	35, 53%
Burmese	5, 7.6%
Domestic longhair	3, 4.5%
British shorthair	3, 4.5%
Ragdoll	3, 4.5%
Siamese	3, 4.5%
Other breeds	14, 21.2%
Body weight (kg)	
Median	4.54
Q1-Q3	4.0–5.17
Min-Max	2.85–9.2
Duration of rivaroxaban dosing (all cats, in days)	
Median	26.5
Q1-Q3	2–60
Min-Max	0–442
Duration of rivaroxaban dosing (survived to discharge, *n* = 45, in days)	
Median	42
Q1-Q3	14–109
Min-Max	1–442
Rivaroxaban dose (mg/day)	
Median	2.5
Q1-Q3	2.5–5.0
Min-Max	1.25–10
Rivaroxaban dose (mg/kg/day)	
Median	0.73
Q1-Q3	0.53–1.0
Min-Max	0.28–1.87
Length of hospital stay (days)	
Median	0
Q1-Q3	0–1
Min-Max	0–2
Outcome	
Survived to discharge home	45, 68.2%
Euthanised	14, 21.2%
Taken home against medical advice	5, 7.6%
Died	2, 3.0%

### 3.2 Rivaroxaban indication and risk factors for thrombosis

**Figure 1:**
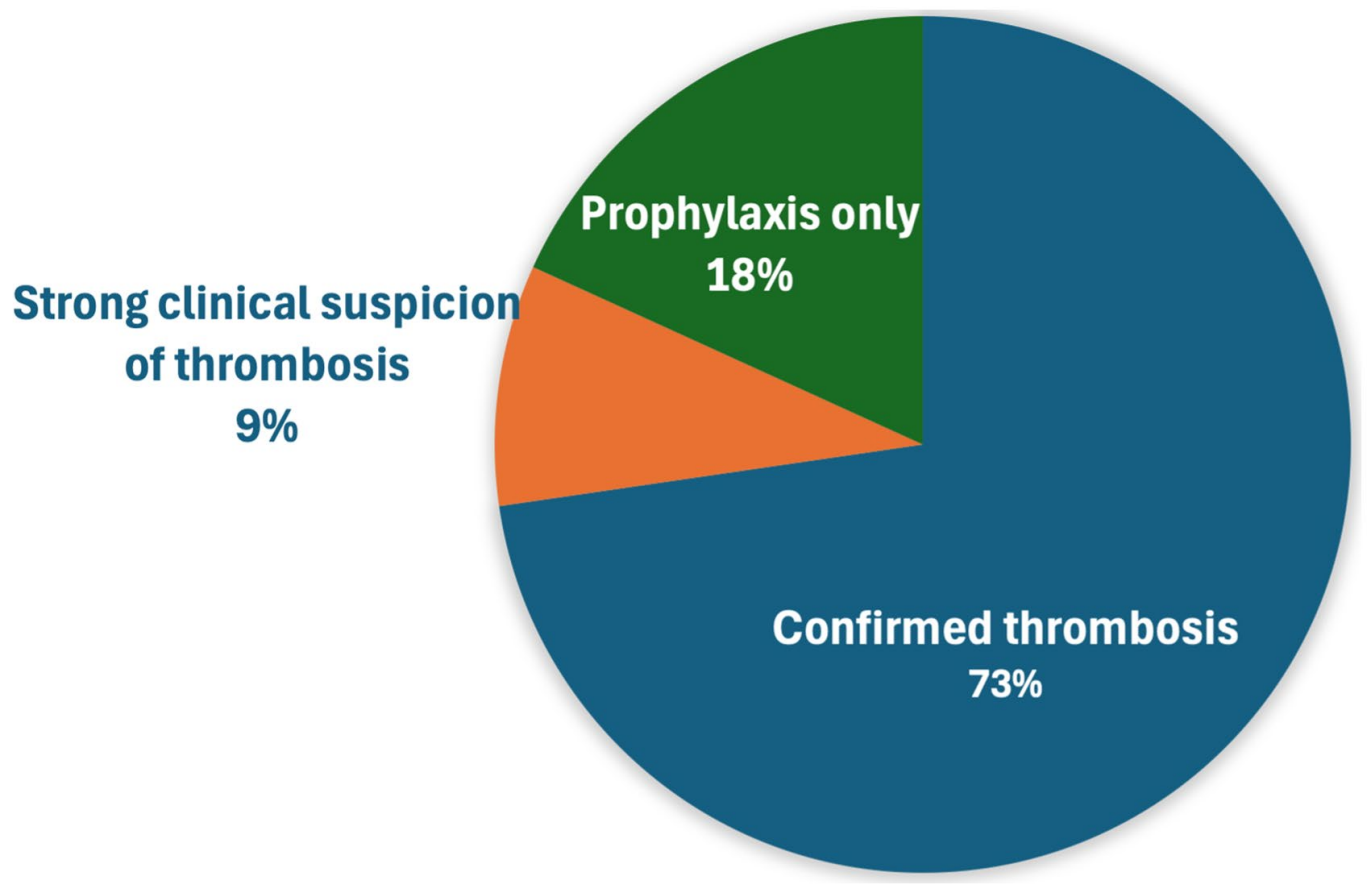
Indications for rivaroxaban administration in 66 cats.

The most common indication for rivaroxaban administration was confirmed thrombosis (48, 72.7%) (Figure 1). Regarding the location of thrombosis, most were arterial in nature, including 25 cats (25/54 with confirmed or suspected thrombosis, 46.3%) with arterial/aortic thromboembolism affecting both pelvic limbs, arterial thrombosis affecting a single limb (16, 29.6%), and cardiac chamber thrombus (7, 13%) most common (Table 2). Other locations of arterial thrombi included cerebrovascular, renal, and spinal arteries, while locations of venous thrombi included portal venous thrombosis (PVT), pulmonary thromboembolism (PTE), and femoral vein thrombosis in a cat after polytrauma (Table 2). Of the seven cats with cardiac chamber thrombus, this was the only thrombus identified in four cats, while one cat each also had ATE affecting both pelvic limbs, ATE affecting three limbs and evidence of CVA, and evidence of single limb ATE and renal artery thrombosis. Segmental spinal infarction with L4-L5 ischemic myelopathy was diagnosed on MRI in one cat.

**Table 2:** Location of thrombosis identified in 54 cats with confirmed or suspected thrombosis that were prescribed rivaroxaban.

Location of thrombus	Arterial (A) or venous (V)	Total number of cases (%)	Number confirmed	Number suspected
Arterial/aortic thromboembolism affecting both pelvic limbs	A	25 (46.3%)	22	3
Arterial thrombosis affecting a single limb	A	16 (29.6%)	16	0
Cardiac chamber thrombus	A	7 (13%)	7	0
Suspected but not MRI confirmed cerebrovascular accident (CVA)	A	5 (9.3%)	5	0
Renal arterial thrombosis, including segmental renal infarct	A	3 (5.6%)	3	0
Portal venous thrombosis	V	2 (3.7%)	2	0
Arterial thromboembolism affecting ≥3 limbs	A	1 (1.9%)	1	0
Pulmonary thromboembolism	V	1 (1.9%)	1	0
Femoral vein thrombosis	V	1 (1.9%)	1	0
Spinal infarction	A	1 (1.9%)	1	0

Note that the total number of cases sums to more than 54 as some cats had thrombi in multiple locations.

Cardiac disease was the most common risk factor for thrombosis (53/66, 80.3%), and two cats had a cardiac arrhythmia in addition to structural heart disease (2, 3%) (Table 3). Other CURATIVE defined risk factors included IMHA in four cats (6.1%), and sepsis in one cat. Four cats (6.1%) had indications for thromboprophylaxis other than a CURATIVE specified risk factor that were nonetheless considered biologically plausible risk factors (1 prophylactic, 3 with thrombi). Three cats (4.5%) had confirmed thrombosis without identification of an underlying cause. A brief description of the IMHA cases, of each of the four cases that had indications for thromboprophylaxis other than a CURATIVE specified risk factor, and the three cats that had confirmed thrombosis in the absence of identified risk factors, is provided in Supplementary material 1.

**Table 3:** Risk factors for thrombosis identified in 66 cats prescribed rivaroxaban.

Risk factor for thrombosis	Classification of risk based on CURATIVE	Number of cases (%)
Cardiac disease	Major	53 (80.3%)
Arrhythmia and structural cardiac disease	Major	2 (3.0%)
IMHA	Other	4 (6.1%)
Sepsis	Other	1 (1.5%)
Biologically plausible but not listed in CURATIVE		4 (6.1%)
None identified but thrombosis confirmed		3 (4.5%)

### 3.3 Rivaroxaban side effects

Four cats (6.2%) had potential rivaroxaban adverse effects including vomiting (1), an acute onset of intracranial signs (1), gastrointestinal haemorrhage (1), and pleural effusion with a haemorrhagic appearance (1). Given the small number of cases, each cat with potential adverse effects is described below.

A 12-year 4-month-old male neutered DSH (5.1 kg) was commenced on rivaroxaban (2.5 mg PO SID) after cardiac chamber thrombus was identified on echocardiogram (in the left atrium and auricle). Rivaroxaban was discontinued after 148 days due to both owner difficulty in giving the medication and vomiting that occurred after medication. This cat was receiving concurrent clopidogrel, pimobendan, and furosemide, so it was unclear which medication(s) was causing the vomiting.

A 10-year-old male neutered exotic shorthair (2.9 kg), diagnosed with HCM based on echocardiogram, was commenced on rivaroxaban (2.5 mg PO SID) and clopidogrel after a thrombus was identified in the left external iliac artery on ultrasound. Bilateral adrenomegaly was also identified on abdominal ultrasound, but endocrine testing was not performed. Three days after commencing rivaroxaban, the cat represented with decerebrate posture and nystagmus, suggestive of a CVA. No further diagnostics were performed to differentiate intracranial thrombosis from haemorrhage, and the cat was euthanised.

A 3.5-year-old female spayed DSH (3.5 kg) was commenced on rivaroxaban (5 mg PO SID), clopidogrel, and aspirin after a diagnosis of bilateral pelvic limb ATE and HCM. Two days after starting thromboprophylaxis, a sudden drop in PCV from 31 to 24% was noted, and melena was evident on day 3, confirming GI haemorrhage. No other location of haemorrhage was identified with bicavitary diagnostic imaging. The cat ultimately developed clinical signs of anaemia and was transfused. Clopidogrel and aspirin were continued without recurrent melena or anaemia, but the cat developed recurrent ATE 1 month later and was euthanised.

The final case with potential rivaroxaban adverse events was an 8-year 8-month MN British shorthair (4.16 kg). He had been commenced on rivaroxaban and clopidogrel after presenting with ATE affecting both pelvic limbs. Initial rivaroxaban dose was 2.5 mg PO SID, later increased to BID, due to suspected thrombosis. Congestive heart failure secondary to HCM was treated with torasemide, spironolactone, and pimobendan. The total duration of rivaroxaban therapy was 219 days. This cat ultimately died of large volume recurrent pleural effusion, which was more haemorrhagic than expected based on gross appearance (fluid PCV not performed) and recurred rapidly despite thoracocentesis.

### 3.4 Additional thromboprophylaxis and concurrent medications

Eight cats (12.1%) received other anticoagulants (subcutaneous dalteparin), during hospitalisation. In six cases, this was prior to rivaroxaban, while one cat each had an anticoagulant commenced concurrent with rivaroxaban or after rivaroxaban. The reason for starting dalteparin first and later transitioning to rivaroxaban as an anticoagulant was not always explained, but in some cases was that the cats were initially inappetant. One cat with bilateral pelvic limb ATE received a single dose of IV thrombolytic (tissue plasminogen activator).

58 of 66 (87.9%) cats also received clopidogrel. Of these, 33 (56.9%) commenced clopidogrel prior to rivaroxaban, 24 (41.4%) commenced clopidogrel concurrently with rivaroxaban, and one case (5.9%), a cat with IMHA, was commenced on clopidogrel after starting rivaroxaban. The addition of clopidogrel in the cat with IMHA was due to clinician concern for ongoing hypercoagulability while on rivaroxaban, specifically, rapid clot formation in blood tubes during collection. Four cats (6.1%) received aspirin concurrent with rivaroxaban and clopidogrel (i.e., three medications for thromboprophylaxis).

Concurrent cardiac medications were common including furosemide (27/66, 40.9%), pimobendan (13, 19.7%), spironolactone (6, 9.1%), benazepril (3, 4.5%), torasemide (2, 3%), and atenolol (2, 3%), and one case each receiving diltiazem, sotalol, hydrochlorothiazide, and combination losartan/hydrochlorothiazide.

### 3.5 Outcome

Forty-five cats (68.2%) survived to hospital discharge, while 14 (21.2%) were euthanised, two (3%) died, and five (7.6%) were taken home against medical advice. Of the non-survivors, non-survival was due to the underlying disease; rivaroxaban was used in 9 of 16 (56.3%), or thromboembolic complications in 7 (43.8%).

## 4 Discussion

This multicentre historical case series describes the clinical use of rivaroxaban in a large population of hospitalised cats, almost doubling the number of rivaroxaban treated cats in the literature. Our findings were consistent with our hypothesis that most hospitalised cats received rivaroxaban after recognition of thrombosis, rather than prophylactically, and that few cats had adverse effects potentially attributable to rivaroxaban. Although the goal of our study was to describe a more heterogenous population of cats than in previous studies, our population was still dominated by cats with cardiac disease and arterial thrombosis. Few cats in this case series had venous thrombosis, consistent with the fairly rare reports of PVT and PTE in cats (30–33), relative to arterial thrombosis.

Consistent with previous publications, most cats received a single daily dose of rivaroxaban. The median dose of rivaroxaban (0.73 mg/kg/d) was in line with the current CURATIVE dosing suggestions (0.5-1 mg/kg/day) (5), but more than 40% of cats received doses outside of the recommended range. There was also greater variation in dosing compared to previous studies where a standard dose of 2.5 mg PO per cat per day was used (23, 25, 26). Numerous factors may account for dose variation including tablet size, lack of data showing benefit of one dosing strategy over another, and clinician caution given limited experience using rivaroxaban in cats. Regarding tablet size, only a single tablet size (15 mg) was stocked at TAHMU, so many cats received a daily dose of 3.75 mg (¼ tablet). The other study hospitals generally wrote prescriptions for their cat patients that were filled by external pharmacies and hence had access to a greater spectrum of tablet sizes (2.5 mg, 10 mg, 15 mg, and 20 mg), as well as a pediatric suspension (1 mg/mL).

Although outside of the scope of this study, it is suspected that clopidogrel is still likely to be used as the first-line drug for thromboprophylaxis in many cats with cardiomyopathy with or without ATE based on results of the FAT CAT study (34) and traditional recommendations for use of antiplatelet drugs for the prevention of arterial thrombosis. Nonetheless, both the CURATIVE and ACVIM guidelines suggest a role for combining clopidogrel with an anticoagulant for cats with ATE (2, 10). In addition, FXa inhibitors are considered standard of care in human beings with non-valvular atrial fibrillation (14), which predisposes to cardiac chamber thrombosis, a condition with similarities to cardiomyopathy-associated cardiac thrombosis in cats.

Most cats in our study received dual thromboprophylaxis with clopidogrel commenced prior to or at the time of rivaroxaban commencement. Our findings are similar to those of Lo et al. who were the first to describe the use of rivaroxaban (2.5 mg PO q24h) as part of dual therapy with clopidogrel (18.75 mg PO q24h), in a historical case series of 32 cats with cardiac disease and/or ATE (23). Indeed, in our study, only 3 of 46 cats with ATE in our study received rivaroxaban without concurrent clopidogrel.

The role of rivaroxaban as a sole antithrombotic in cats with ATE has been the subject of two recent prospective RCTs, also with a focus on cardiac indications (25, 26). The first only included six cats in the rivaroxaban alone group (2.5 mg PO q24 h), compared to 17 cats that received a combination of enoxaparin and clopidogrel. Unfortunately, the reporting of this study did not follow recommended reporting guidelines (35, 36), precluding a complete understanding of the results. In the other relevant RCT (the multicentre, prospective, double-masked SUPERCAT study), 45 cats that had recovered from cardiogenic ATE were randomised to receive either rivaroxaban (2.5 mg PO q24h; *n* = 26) or clopidogrel (18.75 mg PO q24h; *n* = 19) as sole thromboprophylaxis for up to 2 years after the initial ATE (26). Therapy with rivaroxaban or clopidogrel had equivalent impacts on recurrence of thromboembolism (10/26, 39% in the rivaroxaban group, vs. 7/19, 37% in the clopidogrel group), time to recurrence (median 513 days in the rivaroxaban group, vs. 663 days in the clopidogrel group), and survival (26). For comparison, the retrospective study by Lo et al. reported a recurrence rate of ATE while on dual therapy of 16.7% but a median survival time from onset of therapy of only 257 days (Q1-Q3 38–497 days) (23). Given the focus of our study on hospitalised cats, the median duration of rivaroxaban therapy was shorter than in these case series (23) and RCTs (25, 26) where outpatient treatment was studied. Future prospective RCTs should compare rivaroxaban or clopidogrel alone versus dual thromboprophylaxis in cats with cardiac indications, particularly given *ex vivo* evidence of a synergistic inhibitory effect on platelet function and platelet-dependent thrombin generation in cats (37). Future studies could also investigate the risks vs. benefits of triple therapy (rivaroxaban, clopidogrel, and aspirin), given the potential for bleeding, as occurred in one cat reported herein.

Consistent with the primary indication of confirmed arterial thrombosis with underlying cardiac disease, most cats continued therapy until they died, were euthanised, or lost to follow-up. One of the two cats that stopped rivaroxaban at the end of a scheduled course received a 4-day course for femoral vein thrombosis after trauma. The other was commenced on long-term clopidogrel but only a 14-day tapering course of rivaroxaban after cardiogenic ATE. The potential for rebound hypercoagulability and hence the need to taper rivaroxaban prior to discontinuation in cats and dogs is not known (7) and warrants further study.

A low number of cases of potential rivaroxaban adverse effects were evident in this study. The only case with clear evidence of haemorrhage associated with thromboprophylaxis was a cat receiving rivaroxaban in combination with clopidogrel and aspirin, where rivaroxaban discontinuation resolved gastrointestinal haemorrhage. Haemorrhage was a concern in two other cases but could not be differentiated from a CVA in one case or pleural effusion associated with CHF in another case. Nonetheless, our findings were similar to those of other authors where adverse effects appear uncommon (22, 23). Since retrospective studies are not ideal to evaluate drug safety, further prospective studies with a focus on safety and effectiveness of rivaroxaban in hospitalised cats are warranted.

Limitations of our study are related to its retrospective nature. In addition, despite being the largest study of the use of rivaroxaban in cats to date, the sample size is still small, and thus, our findings may not be representative of the broader experience of rivaroxaban use in cats. Data on the duration of rivaroxaban therapy may have been inaccurate due to the assumption that prescribed rivaroxaban was administered as instructed following discharge and the high rate of loss to follow-up. Similarly, documenting adverse effects relied on clinician reporting and thus may underestimate the true incidence. Dose variation, including underdosing of some cases relative to recommendations, may have also led to low rates of reported adverse effects, although a greater proportion of cats were dosed above recommendations than below. No conclusions can be drawn regarding rivaroxaban effectiveness at controlling existing thrombosis or preventing new thrombosis. Not all cats had specialist echocardiograms, and thus, the nature and severity of underlying cardiac disease was not routinely reported.

## 5 Conclusion

Rivaroxaban was well tolerated in a large population of cats in this study, suggesting that it is an appropriate first-line anticoagulant choice in cats with or at risk of thrombosis. Our results provide further support for prospective studies assessing the safety and effectiveness of rivaroxaban in cats, including in combination with clopidogrel. Dual thromboprophylaxis has the potential to improve effectiveness but may increase the risk of haemorrhage. Further research in this field will ultimately facilitate improved levels of evidence and guide updates to relevant clinical practice guidelines.

## Data Availability

The raw data supporting the conclusions of this article will be made available by the authors, without undue reservation.
