# Supplementary material for: Indications and outcomes of rivaroxaban use in cats
Source: Front Vet Sci. 2025 Jun 20;12:1561003. doi: 10.3389/fvets.2025.1561003 (PMC12226864; doi:10.3389/fvets.2025.1561003)
Supplement: Supplementary file 1 [file Table_1.docx]

Supplement 1.

A brief description of the IMHA cases, each of the four cats that had indications for thromboprophylaxis other than a CURATIVE specified risk factor, and the three cats that had confirmed thrombosis in the absence of identified risk factors, is provided.

The four cats with a diagnosis of IMHA had rivaroxaban commenced prophylactically i.e. there was no evidence of thrombosis at the time of commencement. These four cats were also treated with glucocorticoids during hospitalization, two each with prednisolone and dexamethasone, which may have contributed to the clinician’s decision to commence thromboprophylaxis. Two of the four cats with IMHA were also treated with clopidogrel; commenced concurrently in one, and after rivaroxaban in the other. Three of the four cats with IMHA had non-associative IMHA, while the other was considered associative IMHA given the presence of a gastric mass. One cat with IMHA also had evidence of cardiac disease, specifically an auscultable gallop sound and biatrial enlargement on thoracic radiographs, but echocardiography was not performed. This cat also had a hypocoagulable ROTEM prior to commencing rivaroxaban, for which a consumptive coagulopathy was the top differential diagnosis. While all four cats with IMHA survived to discharge, the aforementioned cat with concurrent cardiac disease was suspected to die of a thrombotic event despite rivaroxaban and clopidogrel 26 days after discharge. She had an acute onset of vocalising and open mouth breathing at home, and presented dead on arrival to a local emergency clinic.

The four cats that had indications for thromboprophylaxis other than a CURATIVE specified risk factor are described. Firstly, a 15 month old male neutered Cornish Rex diagnosed with feline infectious peritonitis based on IFA had evidence of portal venous hypertension on abdominal ultrasound with secondary splenic venous distention and tortuosity, reduced hepatopetal flow velocity (12.8cm/sec), and multiple aberrant tortuous vessels in the region of the left renal vein. This cat also had a grade II/VI left parasternal systolic heart murmur, but echocardiography was not performed. Rivaroxaban (1/8^th^ of 15mg tablet PO SID) was commenced due to concern that blood stasis and/or turbulence could cause thrombosis.

An 11 year 8 month old British Shorthair had multifocal thrombosis of the intrahepatic divisions of the portal veins identified on CT. These were incidental; first identified on abdominal ultrasound to investigate lower urinary tract signs, and then further characterised on CT. The reason for thrombosis was listed as biologically plausible because this cat had evidence of liver disease and neoplasia, but the contribution of each was unclear. Regarding liver disease there was marked contrast enhancement of the left hepatic division and caudoventral aspect of the right division in the arterial/portal phase and an ill-defined and poorly contrast enhancing nodule (6 mm diameter) in the left lateral liver lobe. There was also an acquired extrahepatic portosystemic shunt in the left cranial abdomen, with vascular aberrancy described as a nest of multifocal aberrant and tortuous vessels arising from the splenic and left gastric veins, looping at the level of the celiac and cranial mesenteric arteries and then forming a larger single vessel (3.5mm diameter) that entered into the caudal vena cava immediately cranial to the confluence of the left renal vein. Regarding neoplasia the cat also had a large pulmonary mass, and mediastinal lymphadenomegaly identified on CT, although sampling was not pursued. ROTEM was hypocoagulable at the time, suspicious for a consumptive coagulopathy. Both rivaroxaban and clopidogrel were commenced initially, with rivaroxaban continued long-term.

The third cat with a biologically plausible risk factor for thrombosis was a 3 year 4 month old DSH with a thrombus identified in the left femoral vein on CT and ultrasound imaging after polytrauma. There was also swelling of the left pelvic limb that may have contributed to venous stasis and the resultant thrombus. This cat received a four day course of rivaroxaban, and concurrent clopidogrel.

Finally, a 11 year old FS DMH presented with arterial thrombosis causing right pelvic limb paresis. Ventricular ectopy was identified on ECG with right bundle branch morphology, but the heart was structurally normal on echocardiogram performed by a specialist cardiologist. As such this case does not fulfil the CURATIVE defined risk factor of arrhythmia with structural cardiac disease. The cat also had a bronchointerstitial pattern on thoracic radiographs interpreted by a specialist radiologist; these pulmonary parenchymal changes did not improve with diuretic therapy and the cat was lost to follow-up.

The three cats that had confirmed thrombosis in the absence of identified risk factors are described. A 6y 5m FS Burmese cat presented for acute left thoracic limb paresis consistent with arterial thromboembolism. Three days after commencing thromboprophylaxis the cat also developed vestibular signs suggestive of cerebrovascular accident, but MRI was not performed. Echocardiography performed by a specialist cardiologist revealed a structurally normal heart. Abdominal and thoracic CT failed to identify a cause of thrombosis. The only other clinical abnormalities were ocular changes and chronic upper respiratory tract signs attributed to chronic feline herpesvirus infection, and allergic skin disease. The cat was lost to follow-up after 109 days.

A 12 y 9m FS DSH had PVT and PTE identified on abdominal and thoracic CT. Other clinical problems a lower urinary tract infection, bilateral otitis externa, and presumptive inflammatory bowel disease, without protein losing enteropathy, based on abdominal ultrasound, but not confirmed with biopsy. It is unclear if one or more of these diseases predisposed to venous thrombosis. Rivaroxaban and clopidogrel were commenced concurrently, and treatment ongoing at the time of data collection for the study.

A 1 year old MN DMH presented with an acute onset of pelvic limb paralysis consistent with distal aortic thromboembolism. Single doses of rivaroxaban and clopidogrel were administered, but left thoracic limb thrombosis developed in hospital, and the cat was euthanised without further investigation. Underlying cardiac disease was suspected given the signalment and nature of the thrombosis, but not confirmed.
